# Unusual Case of a Proptosed Eye: Isolated Right Maxillary Neurofibroma

**DOI:** 10.1155/2016/4294729

**Published:** 2016-09-28

**Authors:** Darren Yap, Hannah Fox, Julia Addams-Williams

**Affiliations:** Department of ENT, Royal Gwent Hospital, Cardiff Road, Newport NP20 2UB, UK

## Abstract

Neurofibroma is a slow growing benign tumour of the peripheral nerve sheath which is frequently associated with neurofibromatosis type 1 (Prakash et al., 2014). Isolated solitary occurrence of neurofibroma in the maxillary sinus is rare with only 29 reported cases in the literature. We present a rare case of a 70-year-old gentleman who was referred to ENT with a right maxillary sinus neurofibroma with extension into the right inferior orbit. He has significant proptosis, ptosis, and limitation in abduction of the right eye. He has a complicated past history of multiple neurofibromas which were completely excised. Craniofacial MRI shows a large mass filling the right maxillary antrum extending anteriorly into subcutaneous tissue towards nasal ala and posterolaterally into inferior temporal fossa and superiorly into orbit and cavernous sinus involvement. Biopsy of the right maxillary mass revealed cellular spindle cell tumour with wavy collagen bundles within myxoid stroma which is consistent with a neurofibroma. Patient's case was discussed in the skull-base MDT and he has been referred to a specialist center for surgical removal of the neurofibroma with reconstructive surgery. Despite the rarity of this disease, otorhinolaryngologist should consider a possibility of neurofibroma of the paranasal sinuses.

## 1. Introduction

Neurofibroma (NF) is a benign slow growing tumour of the peripheral nerve sheath. It may occur as an isolated sporadic lesion or may be associated with neurofibromatosis type 1, which is also known as von Recklinghausen's disease. Neurofibroma of the maxillary sinus is an exceedingly rare tumour. We report a case of right maxillary sinus neurofibroma and literature review of this rare condition.

## 2. Case

We present a case of 70-year-old gentleman who was referred to the ENT department with a one-year history of right maxillary sinus mass. He presented with significant proptosis, ptosis, and limitation in abduction of the right eye. He also complained of right sided facial pain with no history of epistaxis or nasal congestion.

Patient has a past medical history of multiple neurofibromas which had been excised. He is otherwise fit and healthy.

**Figure 1:**
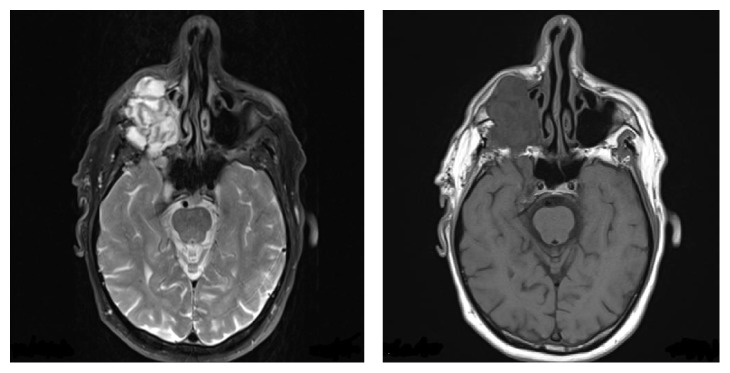
Axial MRI showing lobulated mass in the right maxillary antrum extending anteriorly into the subcutaneous tissue of the cheek and posteriorly into the infratemporal fossa.

**Figure 2:**
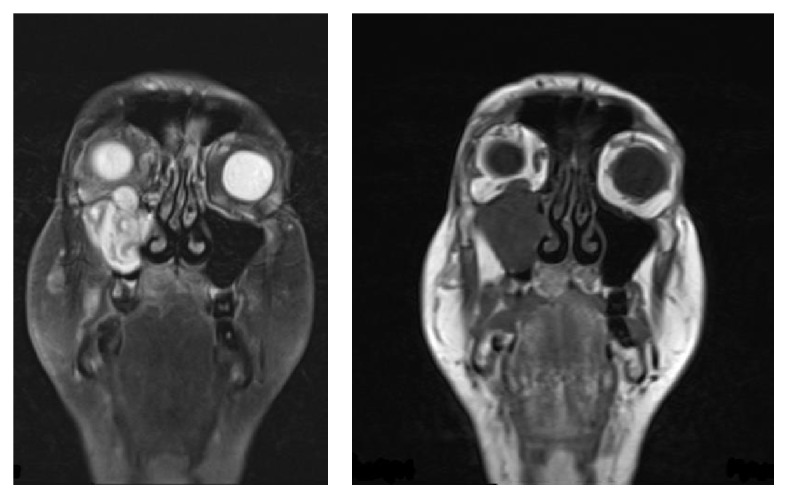
Postgadolinium MRI coronal sequence showing superior extension into the right orbit.

Craniofacial MRI scan has shown a large lesion within the maxillary sinus itself which completely fills and extends into the inferior orbit, cavernous sinus, and pterygopalatine fossa as shown in Figures 1 and 2.

Biopsy of the right maxillary mass revealed cellular spindle cell tumour with wavy collagen bundles within myxoid stroma which is consistent with a neurofibroma. He was treated surgically and currently being followed up annually by the ENT department.

## 3. Discussion

### 3.1. Discussion

Neurofibromas are benign, slow growing, and relatively circumscribed but nonencapsulated peripheral nerve sheath tumours arising from nonmyelinating Schwann cells [1–5]. They can arise as solitary tumours or multiple tumours as a component of neurofibromatosis [2, 4–6]. Neurofibromatosis is not a single entity, but a group of heterogeneous multisystemic neurocutaneous disorders involving both neuroectodermal and mesenchymal derivatives [7]. It is one of the most common hereditary diseases occurring in 1 of every 3000 births [8].

The National Institute of Health (NIH) has defined neurofibromatosis to two distinct types [9, 10]:

1. Peripheral type (NF-I; von Recklinghausen's disease) is associated with changes in the long arm of chromosome number 17 and accounts for over 90% of cases. Major defining features include neurofibromas, cutaneous café au lait macules, and Lisch nodules (pigmented iris hamartomas).
2. Central type (NF-II) is associated with a defect near the center of long arm of chromosome number 22 and is characterized by bilateral acoustic neuromas.

The World Health Organization (WHO) further classifies neurofibromas into dermal and plexiform. Dermal neurofibromas are located mainly in skin areas and composed of single peripheral nerve with soft pedunculated masses of skin in a form of bump. On the other hand, plexiform neurofibromas are composed of many nerve bundles and located mainly within subcutaneous area [3].

Neurofibromas of the nose and paranasal sinuses arise from the ophthalmic and maxillary division of the trigeminal nerve and autonomic plexuses [4, 11, 12]. At present there are only 29 reported cases in literature as shown in Table 1, with only two reported bilateral solitary maxillary sinus neurofibromas and the youngest case being 5 months old [13, 14].

### 3.2. Sign and Symptoms

Symptoms described by patients are nonspecific and depend heavily on the exact location and extension of the lesion [4, 6]. They are thus often clinically silent reaching considerable size in this location before diagnosis [6, 11, 12]. Advanced neurofibromas arising in the maxillary sinus cause pain, epistaxis, nasal obstruction, proptosis, and swelling of the face as described in this case [12, 29].

### 3.3. Imaging

Imaging may not provide a definitive diagnosis; however it plays an important part in assessing, staging, operative planning, and monitoring of the progression of the disease [5].

CT scan may show heterogeneous soft tissue density and destruction of paranasal sinuses dependent on the stage of the disease [6]. Whereas MRI scan usually shows an isotense (lower intensity signal than gray matter) on T1-weighted images and hypertense (higher intensity signal than gray matter) on T2-weighted images [4, 5, 11, 12].

### 3.4. Immunohistochemistry

These lesions may be difficult to distinguish initially as their histology may be similar to other fibrous lesions such as juvenile fibromatosis or fibromyxoma in small biopsy or curettage specimens [12, 14]. Therefore biopsy should include an area of the lesion from deeper portions of the soft tissue swelling, sufficient for macroscopic and microscopic examination and immunohistochemical study [12, 14].

Histopathological examination of neurofibroma typically shows curve indent and ovoid Schwann cell nuclei with spindle cells, wavy nuclei, wavy collagen fibrils, and scatter mast cells embedded in an extended extracellular matrix [1, 4, 12, 17].

Whereas schwannomas show distinctive areas identified as Antoni types A and B, typical palisade pattern of nuclei, encapsulated, solitary, and tumour density is higher compared to neurofibromas, which usually show a mucoid extracellular matrix with only scatter tumour cells [5, 20].

In contrast, malignant peripheral nerve sheath tumours (PNST) are characterized by hypercellularity, “herringbone” fascicular growth pattern, atypical mitotic figures, and pleomorphic tumour cells and nuclei [17, 20].

Immunochemistry can further help to differentiate neurofibromas from schwannomas and malignant peripheral nerve sheath tumours. Neurofibromas show characteristic immunoreactivity with S-100, neurospecific enolase (NSE), and Vimentin [1, 2, 4, 6, 20].

However it is weak and shows focal to patchy reactivity to GFAP, SOX10, and bcl-2 [17].

Malignant PNST tend to show higher percentage of cells positivity and stronger intensity of staining [33].

Calretinin is a calcium-binding protein which is present in a diffuse and strong fashion in schwannoma; aiding the differentiation between neurofibroma and schwannoma as S-100 protein is positive in both tumours.

### 3.5. Differential Diagnosis

NF of the sinonasal tract is difficult and often misdiagnosed. Differential diagnoses in the order of frequency are shown as follows [18, 33].

 Differential diagnosis of sinonasal tract neurofibroma (in order of frequency):

1. Schwannoma
2. Dermatofibrosarcoma protuberans
3. Fibrosarcoma
4. Meningioma
5. Leiomyoma
6. Solitary fibrous tumour
7. Leiomyosarcoma
8. Malignant fibrous histiocytoma
9. Low-grade sinonasal sarcoma with neural and myogenic features
10. Proliferative fasciitis
11. Inflammatory pseudotumour
12. Fibromatosis
13. Fibrous histiocytoma

### 3.6. Treatment

Peripheral nerve tumours are insensitive to radiotherapy and respond poorly to chemotherapy [5, 29]. Hence, early diagnosis and complete surgical excision are the gold standard treatment in symptomatic masses [1, 2, 4, 5, 20].

At present there is no consensus regarding the indication and optimal timing for resection. In general the tumour can be resected if surgery could be performed without risk of damaging vital structures and disfiguring the patient [5].

If surgical resection is not possible, patients are generally managed conservatively with close observation [5]. Although recurrence rate is low, there have been case reports of malignant transformation of these tumours. The transformation of neurofibroma into malignant peripheral nerve sheath tumour has been observed in 2–14% of cases of neurofibromatosis type 1, with a latency period of about 10–20 years. Thus a close followup is warranted [4, 5, 11, 14, 20, 34]. Pain, change in texture, rapid increase in size, and neurological deficits are indications of a malignant transformation within a preexisting neurofibroma [4, 5].

## 4. Conclusion

Despite the rarity of this disease, otorhinolaryngologist should consider a possibility of neurofibroma of the paranasal sinuses.

## Figures and Tables

**Table 1:** Case reports on maxillary neurofibroma.

Number	Year	Author	Age (year)	Gender	Site (maxilla)	Journal
1	2016	Toms et al. [15]	n/a	n/a	Bilateral	Journal of Neurosurgery
2	2015	Warhekar [10]	22	Male	Left	Journal of Clinical and Diagnostic Research
3	2014	Jain et al. [16]	65	Female	Right	Contemporary Clinical Dentistry
4	2014	Azani et al. [17]	52 33 34 66	Female Female Male Female	Right Right Left Left	Head and Neck Pathology (case series)
5	2014	Adnane et al. [6]	14	Male	Left	Journal of Head and Neck Physicians and Surgeons
6	2014	Prakash et al. [1]	60	Female	Right	Research in Otolaryngology
7	2014	Komorski et al. [3]	79	Female	Left	Otolaryngologia Polska
8	2013	Solomon et al. [2]	12	Female	Right	Innovative Journal of Medical and Health Science
9	2012	Rokutanda et al. [12]	41	Male	Right	Journal of Oral and Maxillofacial Surgery, Medicine and Pathology
10	2012	Dalili and Adham [18]	16	Male	Right	Iranian Journal of Radiology
11	2011	Cegarra-Navarro et al. [11]	70	Female	Left	Acta Otorrinolaringol Esp
12	2011	Biswas and Mal [19]	n/a	n/a	Bilateral	Ear, Nose and Throat Journal
13	2011	Thammaiah et al. [7]	2.5	Female	Right	Journal of Paediatric Neurosciences
14	2010	Dass et al. [20]	25	Male	Right	Clinical Rhinology: An International Journal
15	2010	Hachem et al. [5]	10	Female	Right	International Journal of Pediatric Otorhinolaryngology
16	2009	Sharma et al. [14]	5 months	Male	Right	Journal of Indian Society of Pedodontics and Preventive Dentistry
17	2009	Nao et al. [21]	35	Female	Left	Annales d'Otolaryngologie et de Chirurgie Cervico-Faciale
18	2005	Boedeker et al. [4]	25	Male	Left	American Academy of Otolaryngology–Head and Neck Surgery Foundation
19	1997	Poupard and Mintz [22]	n/a	n/a	Solitary neurofibroma of the maxilla	Journal of Oral Maxillofacial Surgery
20	1993	Mori et al. [23]	n/a	n/a	Solitary intraosseous neurofibroma of maxilla	Journal of Oral and Maxillofacial Surgery
21	1988	Tandon and Deka et al. [24]	n/a	n/a	Maxillary neurofibromas	Indian Journal of Otolaryngology
22	1988	Skouteris and Sotereanos [25]	n/a	n/a	n/a	Journal of Oral and Maxillofacial Surgery
23	1982	Brady et al. [26]	n/a	n/a	n/a	Journal of Oral and Maxillofacial Surgery
24	1980	Badger [27]	n/a	n/a	n/a	Journal of the American Dental Association
25	1979	Agarwal et al. [28]	n/a	n/a	n/a	Oral Surgery, Oral Medicine, Oral Pathology
26	1975	Robitaille et al. [29]	45	Male	Right	American Society of Clinical Oncology
27	1975	Toth et al. [30]	n/a	n/a	n/a	Oral Surgery, Oral Medicine, Oral Pathology
28	1970	De Costa et al. [31]	n/a	n/a	n/a	Hospital (Rio de Janeiro, Brazil)
29	1965	Yamazaki et al. [32]	n/a	n/a	n/a	Jibi Inkoka Otolaryngology
